# The facilitating role of phycospheric heterotrophic bacteria in cyanobacterial phosphonate availability and *Microcystis* bloom maintenance

**DOI:** 10.1186/s40168-023-01582-2

**Published:** 2023-06-26

**Authors:** Liang Zhao, Li-Zhou Lin, Ying Zeng, Wen-Kai Teng, Meng-Yun Chen, Jerry J. Brand, Ling-Ling Zheng, Nan-Qin Gan, Yong-Hui Gong, Xin-Yi Li, Jin Lv, Ting Chen, Bo-Ping Han, Li-Rong Song, Wen-Sheng Shu

**Affiliations:** 1 Guangzhou Key Laboratory of Subtropical Biodiversity and Biomonitoring, Guangdong Provincial Key Laboratory of Biotechnology for Plant Development, School of Life Sciences, South China Normal University, Guangzhou, 510631 People’s Republic of China; 2 State Key Laboratory of Freshwater Ecology and Biotechnology, Institute of Hydrobiology, Chinese Academy of Sciences, Wuhan, 430072 People’s Republic of China; 3 Guangdong Provincial Key Laboratory of Microbial Culture Collection and Application, State Key Laboratory of Applied Microbiology Southern China, Institute of Microbiology, Guangdong Academy of Sciences, Guangzhou, 510070 People’s Republic of China; 4 State Key Laboratory of Biocontrol, Guangdong Key Laboratory of Plant Resources, School of Life Sciences, Sun Yat-sen University, Guangzhou, 510275 People’s Republic of China; 5 Department of Molecular Biosciences and the Culture Collection of Algae, University of Texas at Austin, Austin, TX 78712 USA; 6 National Aquatic Biological Resource Center, Institute of Hydrobiology, Chinese Academy of Sciences, Wuhan, People’s Republic of China; 7 Analysis and Testing Center, South China Normal University, Guangzhou, 510631 People’s Republic of China; 8 Institute for Artificial Intelligence and Department of Computer Science and Technology, Tsinghua University, Beijing, 100084 People’s Republic of China; 9 Department of Ecology and Institute of Hydrobiology, Jinan University, Guangzhou, 510632 People’s Republic of China; 10 Guangdong Magigene Biotechnology Co., Ltd., Shenzhen, 518081 People’s Republic of China

**Keywords:** Cyanobacteria, *Microcystis* bloom, Phytoplankton-bacteria interaction, Phosphonate degradation

## Abstract

**Background:**

Phosphonates are the main components in the global phosphorus redox cycle. Little is known about phosphonate metabolism in freshwater ecosystems, although rapid consumption of phosphonates has been observed frequently. Cyanobacteria are often the dominant primary producers in freshwaters; yet, only a few strains of cyanobacteria encode phosphonate-degrading (C-P lyase) gene clusters. The phycosphere is defined as the microenvironment in which extensive phytoplankton and heterotrophic bacteria interactions occur. It has been demonstrated that phytoplankton may recruit phycospheric bacteria based on their own needs. Therefore, the establishment of a phycospheric community rich in phosphonate-degrading-bacteria likely facilitates cyanobacterial proliferation, especially in waters with scarce phosphorus. We characterized the distribution of heterotrophic phosphonate-degrading bacteria in field *Microcystis* bloom samples and in laboratory cyanobacteria “phycospheres” by qPCR and metagenomic analyses. The role of phosphonate-degrading phycospheric bacteria in cyanobacterial proliferation was determined through coculturing of heterotrophic bacteria with an axenic *Microcystis aeruginosa* strain and by metatranscriptomic analysis using field *Microcystis* aggregate samples*.*

**Results:**

Abundant bacteria that carry C-P lyase clusters were identified in plankton samples from freshwater Lakes Dianchi and Taihu during *Microcystis* bloom periods. Metagenomic analysis of 162 non-axenic laboratory strains of cyanobacteria (consortia cultures containing heterotrophic bacteria) showed that 20% (128/647) of high-quality bins from eighty of these consortia encode intact C-P lyase clusters, with an abundance ranging up to nearly 13%. Phycospheric bacterial phosphonate catabolism genes were expressed continually across bloom seasons, as demonstrated through metatranscriptomic analysis using sixteen field *Microcystis* aggregate samples. Coculturing experiments revealed that although *Microcystis* cultures did not catabolize methylphosphonate when axenic, they demonstrated sustained growth when cocultured with phosphonate-utilizing phycospheric bacteria in medium containing methylphosphonate as the sole source of phosphorus.

**Conclusions:**

The recruitment of heterotrophic phosphonate-degrading phycospheric bacteria by cyanobacteria is a hedge against phosphorus scarcity by facilitating phosphonate availability. Cyanobacterial consortia are likely primary contributors to aquatic phosphonate mineralization, thereby facilitating sustained cyanobacterial growth, and even bloom maintenance, in phosphate-deficient waters.

Video Abstract

**Supplementary Information:**

The online version contains supplementary material available at 10.1186/s40168-023-01582-2.

## Background

Organic phosphonates comprise a group of ancient compounds that are characterized by a covalent C–P bond [1, 2]. They dominate the global reduced phosphorus pool (that is, phosphorus with an oxidation number less than +5) [2–7]. Since the inherently stable C–P bond makes the bioavailability of these compounds problematic, the environmental significance of phosphonates was previously considered to be limited [1]. The discovery of C-P lyase, along with alternative substrate-specific pathways such as C–P hydrolase enzyme systems and catalytic oxidative C–P bond cleavage, reveals phosphonate as a significant source of phosphorus for some prokaryotes under phosphorus stress [1–3, 5–8]. For example, the catabolism of phosphonate by the globally important cyanobacterium *Trichodesmium* [9] and other microorganisms [4, 6, 7, 10, 11] occurs through C-P lyase pathways. These are regarded as an adaptation against limited availability of phosphorus from organic sources, thus facilitating optimization of the marine phosphorus cycle. Of the various biogenic phosphonates that have been characterized, methylphosphonate (MPn) is of the greatest interest due to its widespread microbial origin [12, 13] and its role in oxic methanogenesis [10, 11, 14–18].

Pervasive cyanobacteria blooms formed by *Microcystis* are recognized as major threats to freshwater supplies worldwide [19, 20]. Phosphorus limitation is frequently observed in aqueous systems, including eutrophic lakes [21], which may endow adaptive cyanobacteria with an advantage because of their ability to assimilate alternative forms of phosphorus such as phosphonates [9, 11]. Although phosphonate biosynthetic microbes are prevalent in water [8, 13], the occasional phosphonate identification in *Microcystis* aggregates and bloom waters correlates with a rapid consumption of these compounds [22, 23]. However, little is known about phosphonate biotransformation in freshwater ecosystems. Very few unicellular cyanobacteria have been shown to possess a C-P lyase gene cluster and *Microcystis*, in particular, is incapable of utilizing MPn as its sole source of phosphorus [24, 25]. Approximately 6% of cyanobacteria with published genome sequences carry an intact C-P lyase gene cluster, and over 93% of these are filamentous [11, 25]. In contrast, Villarreal-Chiu et al. [8] identified genes for C-P lyase pathways in 16% of bacteria with published genomes.

Cyanobacterial cells in field samples are usually surrounded by abundant heterotrophic bacteria. These phycospheric bacteria utilize organic substrates released by the cyanobacterium [26, 27]. Extensive cyanobacteria-bacterial interactions occur within the phycosphere microenvironment [26]. Metagenomic research and other approaches have profiled structurally and functionally diverse microbial communities within phycospheres [26–29]. It has been demonstrated that the phytoplanktonic phycosphere exerts selective pressure on associated bacteria and establishes microbiomes based on its own demands [27, 30, 31]. The associated heterotrophic bacteria benefit from organic substrates exuded by the cyanobacterium while altering (enhancing or suppressing) the growth of the cyanobacterium [20, 32–36]. These bacteria mainly cooperate through growth factor secretion [37, 38], nutrient regeneration [19, 39], and detoxification [19, 40] and are thought to play a significant role in cyanobacterial proliferation [19, 20, 34–40].

Herein, we describe the occurrence and expression of phosphonate-degrading genes in *Microcystis* blooms of freshwater lakes in the People’s Republic of China, which have undergone intermittent phosphate deficiencies after long periods of water management. This relationship was also demonstrated in many non-axenic laboratory strains of cyanobacterial through metagenomic sequencing. Many of the heterotrophic bacterial strains isolated from cyanobacterial phycospheres were shown to contain phosphonate-degrading gene clusters. Some of these strains facilitated the growth of axenic cultures of *Microcystis* in the presence of MPn as the only source of phosphorus when cultured as consortia.

## Methods

### Genome sequencing, assembly, and annotation

In total, 162 strains of cyanobacteria from the Freshwater Algae Culture Collection at the Institute of Hydrobiology (FACHB), Chinese Academy of Sciences, were selected for examination in this study, 110 of whose genome sequences we have previously published [41] (see Supplementary Dataset 1 for more details). The total genomic DNA of all strains was extracted using the DNeasy Plant Mini Kit (Qiagen). DNA samples were sheared to an average length of 350 bp using an ultrasonicator (Covaris M220). Paired-end libraries were prepared using a DNA Library Prep Kit for Illumina (NEBNext Ultra II). These libraries were then sequenced on the Illumina platform (HiSeq 2000, PE150) at Magigene (Guangdong, China). More than two GB of data were generated for each strain.

Raw sequencing reads were trimmed using Trimmomatic v0.36 [42] to remove adapters and low-quality bases (Q <20). Trimmed reads with more than 5 unidentified nucleotides (N) or lengths less than 50 bp were discarded using custom Perl scripts. The filtered reads of each sample were then assembled separately using the SPAdes v3.13.0 pipeline [43] with the following parameters: --meta; -k 43,63,83,93,101,107. The filtered reads were then mapped back to the assembled contigs using Bowtie2 v2.3.5 [44] to determine coverage values. To obtain the genome sequences of cyanobacteria and bacterial epibionts, metagenomic binning was performed for each sample using MetaBAT v2.12 [45] with default parameters. Measures of quality, including the completeness and contamination of obtained genome drafts (bins), were estimated using CheckM [46]. Only genomes with a completeness higher than 90% and contamination less than 10% were retained for further analysis. Open reading frames (ORFs) of genomes were predicted using Prodigal v2.6.3 [47].

### Identification and phylogenetic analysis of C-P lyase gene clusters

PhnC to PhnM sequences from *Escherichia coli* str. K-12 substr. W3110 (D90227.1) were selected as queries for mining the C-P lyase cluster in phycospheric bacterial bins. A local nucleotide Blast database was constructed with the 647 generated bacterial bins (Supplementary Dataset 2), and then, the Tblastn algorithm [48] was used to identify C-P lyase gene clusters. The organization of the C-P lyase gene cluster in each bin was drawn manually from alignment results (Supplementary Dataset 3; Fig. S1).

To assess potential horizontal gene transfer between cyanobacteria and associated bacteria, a phylogenetic tree of phycospheric bacterial *phnJ* genes (Supplementary Dataset 3) and 151 cyanobacterial *phnJ* genes [25] was constructed using maximum likelihood methods under the GTR + Gamma + Invariant model, with 1000 bootstrap replicates, in MEGA-X [49]. The tree was visualized using iTOL [50]. Since distinct clades for *Pseudomonadales*, *Rhizobiales*, and *Rhodospirillales* were observed in the tree, separate *phnJ* quantitative PCR (qPCR) primers targeting these three groups were designed (Table 1).

**Table 1:** Primer sets used in this study

**Primer**	**Sequence (5′-3′)**	**Efficiency**^**a**^	**Function**
27F	AGAGTTTGATCCTGGCTCAG		For bacterial 16S rDNA amplification, identification, and 16S rDNA standard plasmid construction
1492R	TACGGCTACCTTGTTACGACTT		
515F	GTGNCAGCMGCCGCGGTAA	95.39%	For qPCR of total bacterial 16S rDNA
806R	GGACTAC*N*SGGGTATCTA^b^		
PsudmPJ-F^c^	GAGCGCAAGGCGATGGGCAT		For *Pseudomonas* strains *phnJ* gene amplification and *Pseudomonadales* standard plasmid construction
PsudmPJ-R^c^	ACTCGCCGACGATGCCCAGCAC		
PsudmoPJ-622F^c^	GACGAACAGACCAARCGCATGAT	90.30%	For qPCR of *phnJ* in* Pseudomonadales*
PsudmoPJ-910R^c^	GGTGACGGGTCTGGATCAC		
RhizoPJ-F3^c^	ATCATCCAGACGCGCCACCGCATT		For *Rhizobium* strains *phnJ* gene amplification and *Rhizobiales* standard plasmid construction
RhizoPJ-R3^c^	AGTCGGTGCGCATCAGGAA		
RhizobPJ-153F^c^	ATGCCSATGCCYTATGGSTGGGG	87.29%	For qPCR of *phnJ* in* Rhizobiales*
RhizobPJ-558R^c^	CGACCTTSACCGGRTAGGC		
Methyl-F^c^	AGGACCAGGAATTCGTGCT		For *Methylobacterium* strains *phnJ* gene amplification and *Rhodospirillales* standard plasmid construction
Methyl-R^c^	TGTCCGAGCAGACGAACAT		
RhispiPJ-242F^c^	CCCTATGGYTGGGGCAC	97.66%	For qPCR of *phnJ* in* Rhodospirillales*
RhispiPJ-494R^c^	GGATCGGCACCTGRTAGAC		

^a^Amplification efficiency for each qPCR primer set

^b^One base was modified from G to N (shown in italics) to match the cyanobacterial 16S rDNA sequence

^c^Designed in this study

### Isolation, identification, and *phnJ* amplification of cyanobacterial phycospheric bacteria

Eight non-axenic strains of cyanobacteria were selected for isolation of phosphonate-degrading phycospheric bacteria, including *Anabaena lutea* FACHB-196, *Anabaena* sp. FACHB-418 (also known as *Nostoc* sp. PCC 7120), *Calothrix membranacea* FACHB-236, *Nostoc linckia* FACHB-391, *Nostoc flagelliforme* FACHB-838, *Nostoc* sp. FACHB-973, *Microcystis aeruginosa* FACHB-905, and *Oscillatoria tenuis* FACHB-1052. Each strain with its accompanying heterotrophic bacteria was prepared as a culture in BG-11 medium [51], which was then diluted 10, 100, 1000, and 10,000 times. Then, 100 μL of each dilution was spread on an NBRIP/MPn plate that had been prepared by adding 0.2 mM MPn and 1.5% agarose to phosphorus-free NBRI (National Botanical Research Institute) phosphate growth medium (NBRIP) (per liter: MgCl_2_·6H_2_O, 5 g; MgSO_4_·7H_2_O, 0.25 g; KCl, 0.2 g; (NH_4_)_2_SO_4_, 0.1 g; glucose, 10 g) [52]. Single bacterial colonies were selected after incubation in darkness at 28 ℃ for 1 week. Bacteria were identified using 16S rDNA genes that were amplified with universal 16S rDNA primers 27F and 1492R (Table 1). After the isolates were identified, *phnJ* primers were designed based on *phnJ* genes and their adjacent sequences in bacteria of the same genus listed in the NCBI database. The *phnJ* gene of each isolated bacterium was amplified and cloned into a pMD^TM^ 19-T vector, followed by Sanger sequencing.

### Growth of phycospheric bacteria in different sources of phosphorus

To test the ability of heterotrophic bacteria to utilize different sources of phosphorus, six strains from separate genera were selected for growth experiments, including 236_Mycoba, 391_Methyba4, 838_Rhodoco, 838_Shinella, 905_Psudmo1, and 973_Rhizob3. Six types of media were prepared: LB, P-free NBRIP, P-free NBRIP + 0.2 mM K_2_HPO_4_ (NBRIP/PO_4_^3-^), P-free NBRIP + 0.2 mM MPn (NBRIP/MPn), P-free NBRIP + 0.2 mM 2-aminoethylphosphonic acid (NBRIP/2-AEP), and P-free NBRIP + 0.2 mM glyphosate (NBRIP/glyphosate). Triplicate 60-mL volumes of each of these media in 100 mL flasks were inoculated with axenic colonies from the agar plates. Cultures were incubated with shaking at 175 r.p.m. in darkness at 28 ℃. The samples were taken at 24-h intervals (12-h intervals for cultures growing in LB medium) for optical density readings at 600 nm to monitor growth.

### Genome sequencing of phycospheric strains of bacteria

Genome sequencing using the Illumina platform and assembly were first conducted with the six strains listed above, following procedures previously reported [41]. The 391_Methyba4, 838_Rhodoco, 905_Psudmo1, and 973_Rhizob3 strains were also sequenced with the Nanopore MinION platform (Oxford Nanopore Technologies). After data filtering, both Illumina and Nanopore data were used for genome assembly using Unicycler (https://github.com/rrwick/Unicycler) with default parameters. All the genome assembles were deposited in the NCBI Bioproject database under accession number PRJNA949153.

### Cyanobacterial growth in cocultures of phycospheric bacteria and axenic *Microcystis aeruginosa*

Three strains of phycospheric bacteria, 391_Methyba4, 905_Psudmo1, and 973_Rhizob3, were cultured in LB medium. A 5-mL volume of each cell culture was harvested and washed three times with fresh BG-11 medium while the culture was in log-phase growth. The final pellet was suspended in 1 mL of fresh BG-11 medium and pipetted into 150 mL of axenic *Microcystis aeruginosa* PCC 7806 BG-11 culture. Subculturing continued through several transfers to fresh medium over a 3-month period.

To test whether phycospheric bacteria could sustain the growth of axenic *Microcystis* in medium containing MPn as the sole source of phosphorous, a 10-mL volume of each of these coculture systems was inoculated into 150 mL of P-free BG-11 (BG-11/P^-^, amended with 0.36 mM KCl). These cultures were grown for approximately one month to exhaust their stored phosphorus. Three different media were prepared for these P-starved cells: BG-11/P^-^, BG-11/P^-^ amended with 0.18 mM MPn (BG-11/MPn), and BG-11/P^-^ amended with 0.18 mM orthophosphate (BG-11/PO_4_^3-^). Each phosphorus-depleted culture was inoculated in triplicate into each of these three media. Samples (12-mL volumes) were taken every 4 days. The concentration of cyanobacteria was measured by collecting 2-mL volumes of culture on 47 mm GF/C membranes, followed by acetone extraction of chlorophyll and measuring the chlorophyll *a* concentration (chl *a*) spectroscopically [53]. The phosphorus concentration in each culture was measured using an ammonium molybdate spectrophotometric method [54]. The density of heterotrophic bacteria in each culture was determined by counting colony-forming units on LB plates. One milliliter of culture was also sampled and stored in 1% Lugol’s iodine solution, followed by *Microcystis* cell counting with a hemacytometer. Transcriptomic samples were taken for P-depletion condition (day 0), as well as for MPn (day 4 and day 12) and P-repletion (day 4 and day 12) conditions. Methods for transcriptomic sequencing and data analysis followed standard procedures as previously reported [25].

For measurements of rates of methane liberation in cultures, every 4 days 5-mL volumes of each culture were injected into 25-mL sterile culture flasks equipped with airtight septa. Each flask was then placed back under culture conditions. Methane concentrations were measured by taking 1 mL of gas from the headspace of each vial with a polypropylene syringe after 24 h of incubation and immediately injecting it into a GC 9890A gas chromatograph (Sida, China) equipped with a Porapak Q packed column (2.0 m × 3 mm) [25].

### Field samples of *Microcystis* blooms obtained from Dianchi Lake and Taihu Lake

**Fig. 1:**
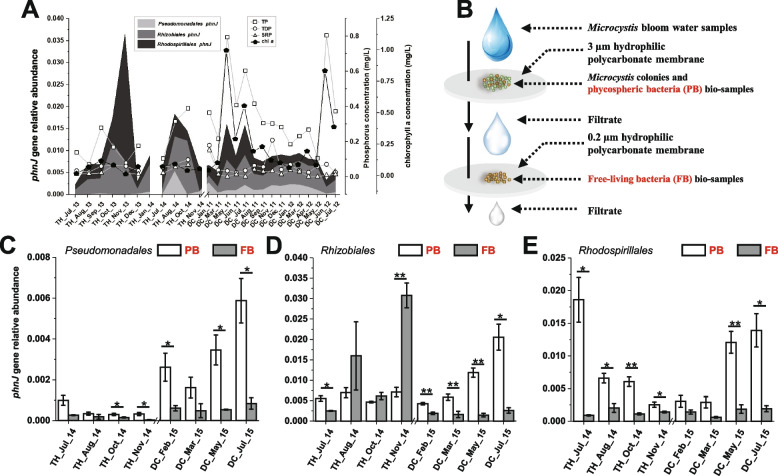
*phnJ* abundances in heterotrophic bacteria of different fractions of bloom samples from two freshwater lakes. **A** Environmental parameters values and relative *phnJ* abundance of three orders of bacteria in Taihu Lake and Dianchi Lake in *Microcystis* blooms at different time periods. **B** Schematic of the protocol used to separate *Microcystis* aggregates containing consortia bacteria from free-living bacteria. **C**–**E** Relative abundance of *phnJ* gene in three orders of phycospheric bacteria and free-living bacteria in Taihu Lake and Dianchi Lake at different times. *p < 0.05; **p < 0.01

Samples of water containing *Microcystis* blooms were taken monthly from site D24 (24.96°N, 102.65°E) in Dianchi Lake from January 2011 to July 2012, and from February 2015 to July 2015. Samples of water were taken monthly from site N2 (31.41°N, 120.19°E) in Taihu Lake during *Microcystis* bloom seasons (from July 2013 to January 2014 and from July 2014 to November 2014). All samples were collected 0.5 m under the surface. The biomass of each sample was collected by filtering bloom water through a 0.2-µm hydrophilic polycarbonate membrane. For the preparation of eight size-graded samples, *Microcystis* colonies and phycospheric bacteria biosamples were first generated using a 3-µm hydrophilic polycarbonate membrane. Free-living bacteria biosamples in the filtrates were then collected by filtering through a 0.2-µm hydrophilic polycarbonate membrane (Fig. 1B). Identical membranes containing trapped biomass were stored at −80 ℃ for later analysis. DNA was extracted from the biomass retained on filters, using the FastDNA™ SPIN kit for soil (MP Biomedicals).

Methods for measuring the values of various environmental parameters in lake water, including water temperature (WT), dissolved oxygen (DO), pH, chl *a*, total phosphorus (TP), total dissolved phosphorus (TDP), soluble reactive phosphorus (SRP), total nitrogen (TN), and total dissolved nitrogen (TDN), were as previously reported by Wu et al. [54]. Detailed sample information and environmental parameter values are provided in Supplementary Dataset 4.

### Quantification of *phnJ* gene abundance in field samples from Dianchi Lake and Taihu Lake

Quantitative PCR standard plasmids were constructed with genomic DNA from 905_Psudmo1 (including both 16S rDNA and a PsudmoPJ plasmid), 973_Rhizob3 (for RhizobPJ plasmid), and Taihu Lake DNA (for RhispiPJ plasmid) (Fig. S2). Standard procedures for plasmid construction and qPCR assays were followed [25]. The relative abundance of *phnJ* was calculated separately for each order of bacteria as the ratio of the abundance of *phnJ* to the abundance total 16S rDNA.

### Taihu Lake metatranscriptomic data processing and analysis

Metatranscriptome raw data that have been deposited into the NCBI database by Zhu et al. [55] with an accession number of PRJNA664620 were utilized in this study. This dataset covers 16 cyanobacterial aggregate samples that were collected from Taihu Lake from April 2015 to February 2016.

The downloaded raw data were trimmed using Trimmomatic (v0.36) [42] to generate clean data. Clean reads were then mapped to the NCBI Rfam database to remove rRNA sequences, using Bowtie2 (v2.33) [44]. The remaining mRNA sequences were assembled de novo with Trinity (v2.4.0) [56], using default parameters, followed by gene prediction with MetaProdigal (version 2.6.3) [57]. The genes were then clustered with MMseqs software [58] using the parameters -e 0.001 --min-seq-id 0.95 -c 0.90 to generate catalogs of non-redundant genes (unigenes). The FPKM (fragments per kilobase per million) value for each unigene in each sample was calculated to estimate gene expression activity using bowtie2 (v2.33) and corset (v1.06) [59].

Genes involved in phosphonate metabolism and methane production were annotated using two methods. For proteins with Pfam numbers, profile Hidden Markov Models (pHMM) were downloaded from https://pfam-legacy.xfam.org, including AepX (PF13343), PafA (PF01663), PepM (PF13714), PhnA (PF01663), PhnD (PF12974), PhnG (PF06754), PhnH (PF05845), PhnI (PF05861), PhnJ (PF06007), PhnX (PF13419), PhoA (PF00245), PhoD (PF09423), PhoH (PF02562), PhoX (PF05787), PmoA (PF14100), PmoB (PF04744), and PmoC (PF04896). The hmmsearch function was then used to identify these proteins using HMMER (v3.3.2) (http://hmmer.org/) with cutoff *E* value < 1E−30. Diamond [60] in EggNOG-mapper (v2.0.1) [61] was employed for the annotation of the remaining proteins (stringency 1E-60). AepX and PafA were validated by phylogenetic tree construction with data from Murphy et al. [62] and Lidbury et al. [63], respectively. MPn-specific PhnY* and PhnZ were identified following published method [64].

## Results

### The widespread occurrence and expression of phosphonate-degrading genes in *Microcystis* bloom samples from Dianchi Lake and Taihu Lake

Genes that encode C-P lyase (*phnJ*) were identified in three selected orders of heterotrophic bacteria in twenty-six *Microcystis* bloom water samples from both lakes (Fig. 1). Genes from *Rhizobiales* and *Rhodospirillales* were especially dominant. A peak in total *phnJ* gene abundance was observed in November 2013 in Taihu Lake, correlating with a sharp drop of TP, SRP, and chl *a* concentrations (Fig. 1A). Peaks appeared in May and July 2011 for Dianchi Lake, while a small decay of SRP but dramatic rise in TP and chl *a* were observed (Fig. 1A). The abundance of *phnJ* genes was quantified separately in phycospheric bacteria and the free-living fraction (Fig. 1B). In the majority (16/24) of analyzed samples, the relative abundances of *phnJ* in the fraction containing phycospheric bacteria were significantly higher than in the fraction containing only free-living bacteria (Fig. 1C–E).

**Fig. 2:**
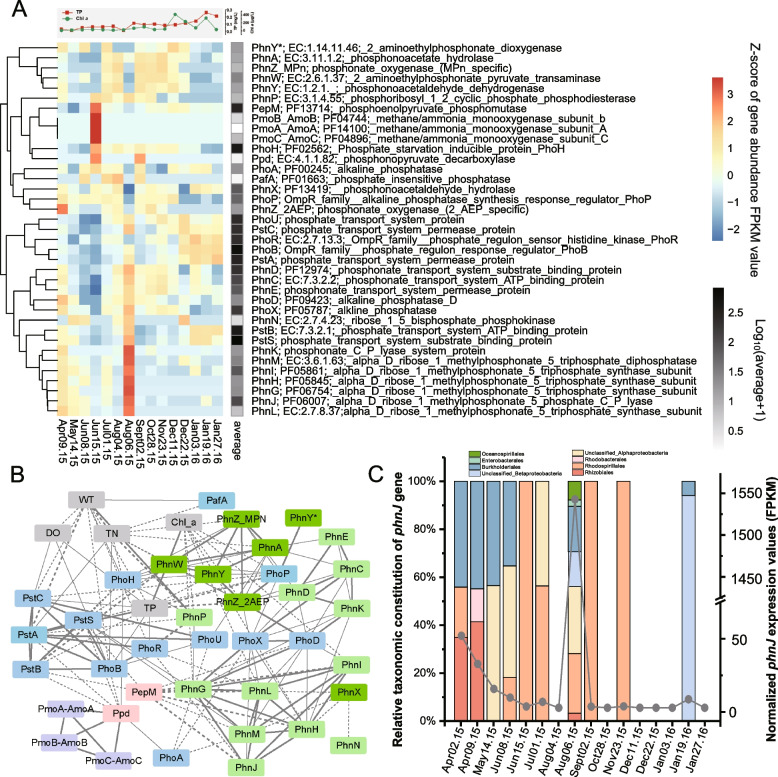
Metatranscriptomic analysis and environmental relationships of genes pertaining to phosphonate metabolism and methane production. **A** Standardized abundance profile (*z* score) for each functional gene. **B** Network analysis between each selected cluster of function and environmental parameter based on Spearman’s correlations. A connection indicates a strong (Spearman’s |*r*|>0.5) and significant (false discovery rate-corrected *p*<0.01) correlation. The thickness of a connection between two nodes represents the value of Spearman’s coefficient. Solid lines indicate positive correlations; dotted lines indicate negative correlations. The color of each node represents a specific category: gray = environmental parameter; purple = genes pertaining to methane metabolism; pink = phosphonate biosynthesis related genes; light green = genes from the C-P lyase gene cluster; dark green = other phosphonate catabolism-related genes; blue = phosphate response genes. **C** Taxonomic constitution and expression values of identified *phnJ* genes across the sampling period

The expression patterns of genes pertaining to phosphonate metabolism and methane production in Taihu Lake during 2015 and 2016 are illustrated in Fig. 2A. The expression of methane oxidation genes (*pmoA-C*) was observed only in the sample from 15 June 2015 during a bloom. No transcription of methyl-coenzyme M reductase genes was observed in this dataset. All genes for C-P lyase clusters except for *phnF* were identified, and *phnC-D* was expressed continuously across the sampling period (Fig. 2A). The expression of all C-P lyase cluster genes peaked on the 6th of August 2015, when a dramatic increase in TP and chl *a* occurred (Fig. 2A).

Genes for other phosphonate biodegradation pathways, including *phnA*, *phnW*, *phnY*, *phnX*, phnY^*^, and *phnZ*, were also identified in most samples, suggesting their role in *Microcystis* blooms. Two unigenes encoding phosphate-insensitive phosphatase PafA were identified (Fig. S3A), while no homologous phosphate-insensitive 2-AEP transporter AepX was annotated (Fig. S3B) in our datasets. No phnY* unigene was validated as MPn-specific (Fig. S4A), and three of six identified *phnZ* unigenes were MPn-specific (Fig. S4B). The expression of C-P lyase cluster genes was positively correlated with the expression of *phoD and phoX*, which were all strongly induced by phosphorus depletion (Fig. 2B). *phnJ* genes occurred primarily in members of *Rhodospirillales*, *Burkholderiales*, and unclassified Alphaproterobacteria (Fig. 2C).

### The widespread occurrence of C-P lyase gene clusters in phycospheric bacteria and their phylogenetics

**Fig. 3:**
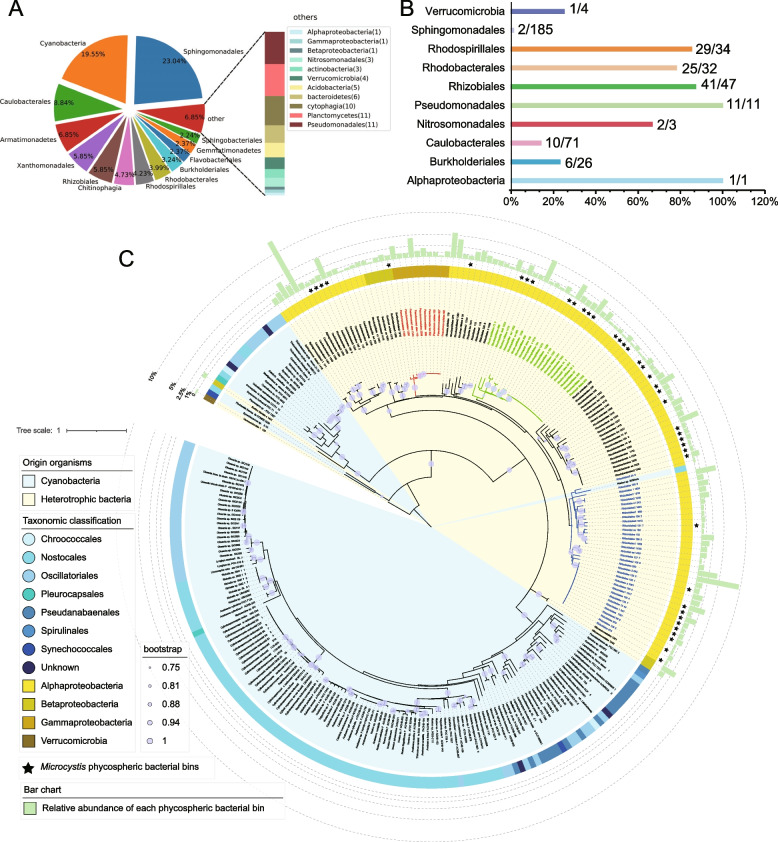
Occurrence and phylogeny of C-P lyase clusters in cyanobacterial phycospheres. **A** The taxonomic constitution of 804 high-quality bins; **B** C-P lyase gene clusters in ten identified orders of bacteria (groups); **C** A maximum likelihood phylogenetic tree of *phnJ* genes from phycospheric heterotrophic bacterial bins and cyanobacteria. A total of 128 *phnJ* genes from 647 phycospheric heterotrophic bacterial bins and 151 cyanobacterial *phnJ* genes [25] were used in tree construction. Clades selected for the design of primer set are shown as red (*Pseudomonadales*), green (*Rhodospirillales*), and blue (*Rhizobiales*) branches. Rings from outer to inner are (1) the relative abundance of each phycospheric bacterial bin in each strain sequencing project, (2) bins generated from *Microcystis* sequencing, and (3) the taxonomic classification of cyanobacterial or heterotrophic bacterial strain for each *phnJ* gene

To investigate the role of phosphonate-degrading bacteria during cyanobacteria proliferation, the phycospheres of 162 non-axenic cyanobacterial strains from the FACHB were analyzed using a metagenomic approach (Supplementary Dataset 1). A total of 804 bins with a completeness higher than 90% and contamination less than 10% were generated, including 157 cyanobacteria bins and 647 heterotrophic bacteria bins (Fig. 3A, Supplementary Dataset 1 and Dataset 2). The number of distinct members of one order of heterotrophic bacteria, *Sphingomonadales* (185 bins) exceeded the total number of cyanobacterial bins (157 bins) (Fig. 3A; Supplementary Dataset 2). Other prominent taxa of bacteria included members of *Caulobacterales* (71 bins), *Armatimonadetes* (55 bins), *Xanthomonadales* (47 bins), *Rhizobiales* (47 bins), *Chitinophagia* (38 bins), *Rhodospirillales* (34 bins), *Rhodobacterales* (32 bins), and *Burkholderiales* (26 bins) (Fig. 3A).

One hundred twenty-eight bacterial bins from ten taxa were demonstrated to encode intact C-P lyase gene clusters, including 41 bins of *Rhizobiales*, 29 bins of *Rhodospirillales*, and 25 bins of *Rhodobacterales* (Fig. 3B; Supplementary Dataset 3). All eleven *Pseudomonadales* bins carried a C-P lyase gene cluster, and the abundance percentages for *Rhizobiales*, *Rhodospirillales*, and *Rhodobacterales* were 87.23%, 85.29%, and 78.13%, respectively (Fig. 3B; Supplementary Dataset 3). In contrast, C-P lyase gene clusters were identified in only two out of 185 *Sphingomonadales* bins. Sequences of each C-P lyase gene cluster were deposited in NCBI with accession numbers from OK646139 to OK646317. The sizes of the C-P lyase gene clusters ranged from 8 to 19 kb, a consequence of frequent phosphonate transporter gene duplication and accessory gene loss or gain within *phnG-M*. The organizations of C-P lyase gene clusters in *Pseudomonadales* bins were highly conserved (Fig. S1). A large portion of *Rhizobiales* bins possessed a similar gene architecture, as did the bins of *Rhodospirillales*. The C-P lyase gene clusters in *Caulobacterales* and *Rhodobacterales* bins were less conserved in both cluster size and organization (Fig. S1).

Several distinct heterotrophic bacterial clades were clustered, as shown in a *phnJ* phylogenetic tree, which had a long genetic distance from cyanobacterial clades (Fig. 3C). A mixed clade of cyanobacteria and heterotrophic bacteria was also seen at the root of the phylogenetic tree. Nearly half of the sequenced cyanobacterial strains (80/162) recruited C-P lyase-encoding bacteria in their phycosphere, with an abundance ranging from 0.21% to nearly 13% (Fig. 3C). Forty-three *Microcystis* phycospheric bacterial bins that contained C-P lyase gene clusters were not well grouped, but were distributed among different bacterial clades. qPCR primers targeted *Pseudomonadales*, *Rhizobiales*, and *Rhodospirillales* clades, but failed to detect *Burkholderiales*, *Caulobacteriales*, and *Rhodobacterales* clades (Table 1; Fig. S2).

### Phosphonate utilization by phycospheric bacteria isolates

Twenty-two strains of MPn-catabolizing phycospheric bacteria were isolated from eight non-axenic cyanobacterial cultures, including eleven strains of *Pseudomonas*, four strains of *Methylobacterium* and three strains of *Rhizobium* (Supplementary Dataset 5). The *phnJ* genes were amplified from each of these strains with genus-specific primer sets.

**Fig. 4:**
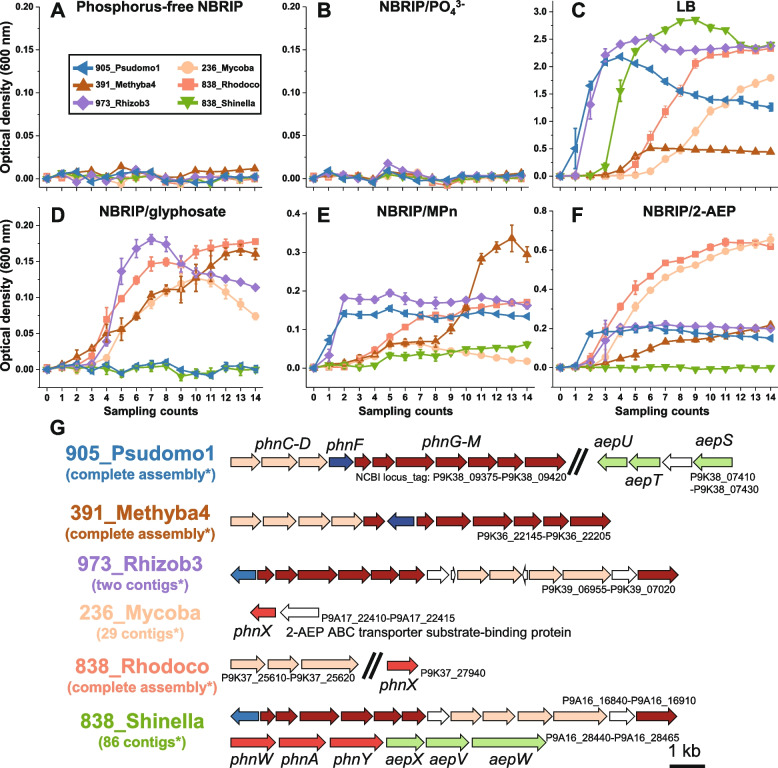
Growth responses of six phycospheric strains of bacteria in media containing different sources of phosphorus. **A**–**F** Relative cell densities. Cell density measurements of bacteria that grew in LB medium were taken at 12-h time intervals; all others were measured at 24-h time intervals. **G** The contents of genes involved in phosphonate catabolism in the genomes of six phycospheric bacterial strains

The growth of six phycospheric heterotrophic strains of bacteria, each cultured in six different media with different phosphorus sources was tested (Fig. 4). LB culture medium was preferred by all strains, while they barely survived in P-free NBRIP medium or in medium containing K_2_HPO_4_ as their sole source of P (Fig. 4A–C). All six strains demonstrated pronounced growth when the culture medium included a phosphonate compound, but no added phosphate. 973_Rhizob3, 391_Methyba4, and 838_Rhodoco thrived in all three phosphonates. 905_Psudmo1 grew well in both NBRIP/MPn and NBRIP/2-AEP medium, but could not utilize glyphosate. Only slight growth was observed when 236_Mycoba was grown in NBRIP/MPn medium, but it reached much higher culture density when grown in NBRIP/2-AEP or NBRIP/glyphosate medium (Fig. 4D–F).

The genome sequences of these strains revealed diverse phosphonate catabolism genes (Fig. 4G). A C-P lyase gene cluster was found in four of six strains (Fig. 4G). *phnX* was the only phosphonate degradation component seen in 236_Mycoba and 838_Rhodoco. Although 838_Shinella could barely utilize 2-AEP (Fig. 4F), a 2-AEP-specific pathway (*phnWAY*) was identified in its genome. In consideration of the fact that all six strains encode intact transport systems PstSCAB and PitA, it is not understood why these strains did not proliferate in NBRIP/K_2_HPO_4_ medium.

### The role of phycospheric bacteria in the utilization of MPn

**Fig. 5:**
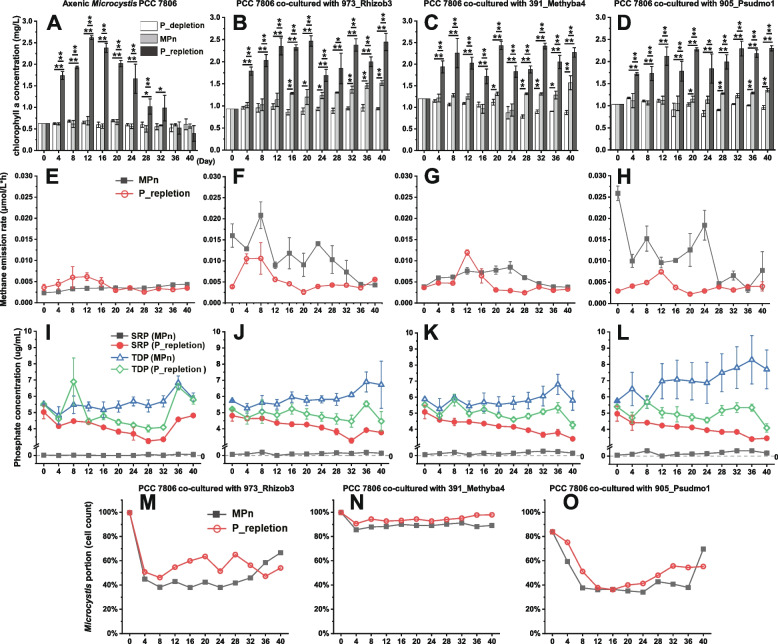
Physiological response of *Microcystis* PCC 7806 grown axenically and in coculture with several different strains of heterotrophic bacteria. **A**–**D** Growth dynamics of cultures grown in media containing three different compositions of phosphorous; **E**–**H** Methane emission dynamics in cultures grown in MPn or P-repletion medium; **I**–**L** Changes in phosphorus concentrations in cultures of four systems grown in MPn or P-repletion medium; **M**–**O** Percentage of total cells (*Microcystis* plus heterotrophic bacteria) that are *Microcystis* in co-cultures grown in MPn or P-repletion medium. *p < 0.05; **p < 0.01

After combining and co-culturing bacteria and axenic *Microcystis* PCC 7806 to obtain stable growth, we investigated whether these organisms individually or in coculture would proliferate in MPn as their sole source of phosphorus. Axenic PCC 7806 cultures did not grow in BG-11/MPn medium (Fig. 5A). In contrast, *Microcystis* cultured in MPn as the sole source of phosphorus grew in the presence of all three tested strains of heterotrophic bacteria (Fig. 5B–D). Although inorganic phosphate was the preferred source of phosphorus for *Microcystis*, MPn served as an acceptable substitute in the absence of phosphate, apparently via consortium bacteria (compare Fig. 4).

The addition of MPn increased the rate of methane production in cocultures (Fig. 5F–H), although low levels of methane emissions occurred in all cultures of *Microcystis* (Fig. 5E–H). All three cocultures grown in the presence of MPn released a small amount of SRP (Fig. 5I–L). *Microcystis* and phycospheric bacteria densities were measured during the growth of each culture. The addition of either MPn or phosphate stimulated the rapid growth of heterotrophic bacteria, which was reflected in a sharp drop in the ratio of *Microcystis* to heterotrophic bacteria by day 4 (Fig. 5M–O). The fraction of *Microcystis* in cocultures in the P-replete medium generally exceeded the ratio when these cocultures were grown in MPn medium (Fig. 5M–O).

**Fig. 6:**
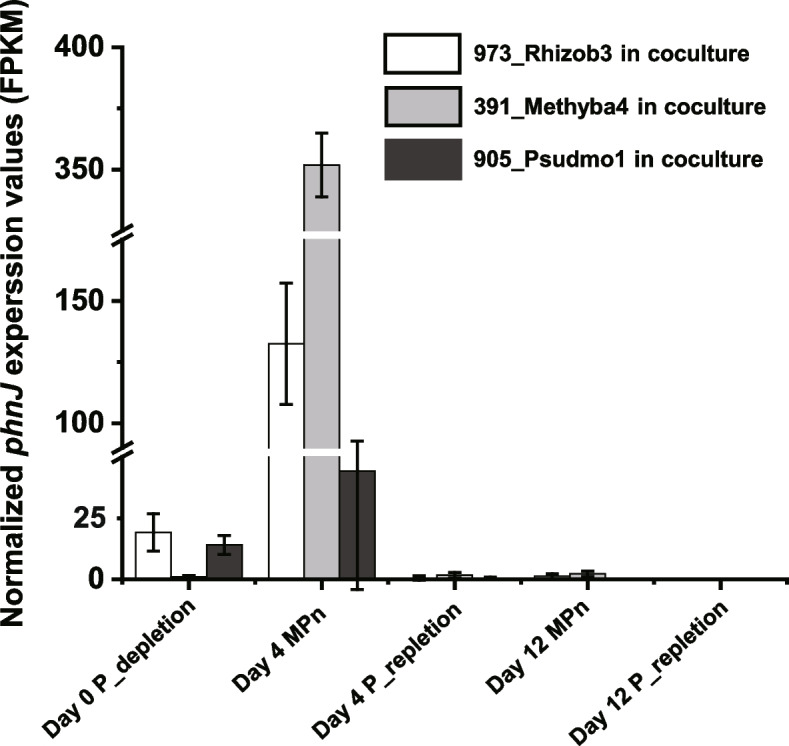
Expression levels of *phnJ* genes in three *Microcystis*-heterotrophic bacteria cocultures containing three different phosphorous compositions

The expression dynamics of *phnJ* genes in each coculture revealed a significant role of phycospheric bacteria in MPn utilization. The expression of *phnJ* genes was slight under P-depletion condition, but was accelerated dramatically upon MPn addition (Fig. 6). In P-replete medium, the expression of *phnJ* was almost completely switched off (Fig. 6).

## Discussion

Important ecological roles of phosphonates in marine environments have been demonstrated through recent studies [11, 24, 62, 63, 65, 66], but little is known about their functions in freshwater. This work demonstrates the ability of freshwater phycospheric bacteria to mineralize phosphonate, while releasing a proportion of bioavailable inorganic phosphate for the associated cyanobacteria. Phosphonate thereby is able to support the growth of phosphorus-starved cyanobacteria that do not contain phosphonate-catabolizing gene elements.

### The role of phosphonate in the freshwater phosphorus cycle

The most prevalent microbial strategy against extracellular inorganic orthophosphate (Pi) deficiency is its generation through extracellular phosphatases [67, 68]. Organic phosphates can be scavenged quickly after they have been liberated into water in the form of DNA or other organic biomolecules [67]. Considering the extensive non-biogenic and biogenic sources of aquatic phosphonate, as well as the inherent stability of the C-P bond, the concentration of phosphonates in natural bodies of water might be expected to be high. However, the occurrence and concentration of phosphonates in oceanographic surveys [4] and freshwater phosphorus contents [22, 23] are lower than expected (Supplementary Dataset 6, Fig. S5). These observations suggest the existence of phosphonate scavenging mechanisms. The frequent presence of *phnJ* genes in freshwater ecosystems may provide an explanation. For example, the rapid dissipation of glyphosate, a widely used artificial phosphonate herbicide, and its derivate AMPA, was correlated with phytoplankton proliferation in Greifensee Lake, and both cyanobacterial and heterotrophic bacterial C-P lyase gene clusters were identified in lake samples [69]. Yao et al. [18] identified *phnJ* homologs in 18 out of 22 examined freshwater lakes by analyzing metagenomic data. We amplified *phnJ* genes from the twenty-six collected field samples (Fig. 1A). The relative abundance of *phnJ* genes in three orders of heterotrophic bacteria in water samples obtained from Dianchi Lake and Taihu Lake (Fig. 1A) was comparable to previous reports of total bacterial *phnJ* gene abundances [66]. The diverse *phnJ* gene distributions among the taxa identified in *Microcystis* aggregates (Fig. 2C) suggests that the three primer sets used to identify *phnJ* may yield an underestimation of total *phnJ* abundance in Taihu Lake.

The metatranscriptomic results provide a detailed landscape of phosphonate utilization by cyanobacterial consortia. The continuous expression of genes encoding the PhoR/PhoB two-component system, as well as genes encoding a PstABCS system and alkaline phosphatase, suggests a shortage of extracellular Pi in Taihu Lake, which is consistent with field observations [21]. Thus, the identification of *phnC-M* genes in our dataset may be expected, since the C-P lyase gene cluster has been shown to be directly controlled by the PhoR/PhoB two-component system [1, 68]. Likewise, in marine systems, *phnJ* and *phnD* gene abundances and expression levels correlate negatively with Pi concentrations [62, 66]. However, in recent transcriptional studies, Teikari et al. [11] and Murphy et al. [70] suggested that the expression of *phnG-M* and other phosphonate genes might be activated only in the presence of a suitable phosphonate substrate, based on the bipartite structure of the C-P lyase gene cluster. That proposal is supported by results seen in our coculture systems (Fig. 6). Among known phosphonate catabolic pathways, genes for a non-specific C–P lyase system (*phnC-M*) [1], a 2-AEP phosphonate oxygenase system (phnY^*^) [71] and a 2-AEP-specific phosphate insensitive phosphonoacetate hydrolase system (*phnWAY*) [65], as well as several MPn-specific and 2-AEP-specific phosphonate oxygenase gene (*phnZ*) (Fig. S4B), were identified in our metatranscriptomic datasets. In summary, the mineralization of phosphonates, especially biogenic phosphonates MPn and 2-AEP, serves as a constant and substantial source of phosphorus in Taihu Lake during the cyanobacterial bloom period, which may help explain the persistence and dominance of *Microcystis* blooms.

### Cyanobacteria phosphonate utilization: the role of phycospheric bacteria

Given their long time period of cultivation in phosphate-enriched cultures, it might be unexpected that laboratory-grown cyanobacteria cultures would support abundant C-P lyase-encoding bacteria. However, the abundance of *phnJ* in phycospheric and free-living bacteria (Fig. 1C–E) indicates the presence of active C-P lyase-encoding heterotrophic bacteria that are recruited by cyanobacteria upon phosphate depletion.

According to several surveys, strains of cyanobacteria that carry genes for phosphonate catabolic pathways are relatively rare [11, 24, 25]. For example, our previous genomic study showed that only approximately 6% of cyanobacteria have an intact C-P lyase gene cluster and most of those with C-P lyase genes (141/151 = 93.4%) are filamentous [25], although a few unicellular cyanobacteria contain phnY^*^Z pathways [24, 72]. In contrast, approximately 40% of heterotrophic bacteria encode one or more phosphonate catabolism pathways, as estimated by Villarreal-Chiu et al. [8]. According to our examination, the percentage of phycospheric strains of bacteria that carry a C-P lyase gene cluster (128/647 = 19.8%) is similar to that of marine strains (23.2%) [8]. The dominance of phycospheric Proteobacteria that carry a C-P lyase (127/128) is also consistent with the results of other studies [8, 24]. Our observation that genes for phosphonate catabolism enzymes are found mostly within heterotrophic bacteria in *Microcystis* bloom-waters strongly suggests a major role of these bacteria in phosphonate utilization by cyanobacteria consortia.

Coculture experiments demonstrate indirect *Microcystis* phosphonate utilization via consortia bacteria. The bacteria-generated phosphate significantly supports the proliferation of phosphorus-depleted cyanobacteria (Fig. 5B–D). Phosphorus transfer between cyanobacteria and attached bacteria has long been observed [73–76]. Since C-P lyase is known to function intracellularly [1, 2], cyanobacterial growth in our coculture systems may be attributed to one or both of two potential mechanisms: (1) MPn is degraded within heterotrophic bacteria cells, followed by the direct release of Pi into the phycosphere [62, 74, 75]; (2) heterotrophic bacteria grow rapidly after MPn is added, followed by culture decline and phosphate release into the phycosphere environment as cells lyse. The latter mechanism is suggested by the SRP concentration (Fig. 5J–L) and heterotrophic bacteria percentages (Fig. 5M–O) in cocultures grown in media containing MPn. In either case, the energy that sustains cell growth and facilitates phosphorus mineralization by commensal heterotrophic bacteria mainly comes from their associated cyanobacteria [26]. In consideration of the quick drop of heterotrophic bacteria cell densities as cultures age (Fig. 5M, O)*, Microcystis* proliferation in cocultures as described here may occur via either or both of these mechanisms, perhaps accompanied by other phytoplankton-bacteria interactions that vary with growth stage [26, 77, 78].

### The potential ecological significance of phosphonate utilization by cyanobacterial consortia

As the dominant form of reduced organic phosphorus, biogenic phosphonate represents a significant portion of the aquatic organic phosphorus pool and the global phosphorus redox cycle [2–5, 24, 79]. Relative to the more bioavailable aminophosphonates, alkylphosphonates are regarded as recalcitrant to oxidation and might be expected to accumulate in bodies of water [15, 16]. However, the typically absent or low concentrations of phosphonates observed in field surveys [22, 23] (Supplementary Dataset 6, Fig. S5) indicate the exhaustive catabolism of most phosphonates. Isolated phycospheric bacteria efficiently catabolize both alkylphosphonates and aminophosphonates [18] (Fig. 4). Thus, cyanobacteria and the bacteria with which they associate may together be core players in the mineralization and utilization of phosphonates, thereby driving the global phosphorus redox cycle in aquatic ecosystems.

Biogenic phosphonates are incorporated into membrane lipids and glycans as head or side groups, but little is known about their ecological role [1, 2]. Although no *pepM* genes were present in most of our phycospheric bins, those that were identified were expressed continuously, as seen in the cyanobacterial consortia metatranscriptomic data (Fig. 2A). The observation that phosphonates are more frequently and abundantly detected in the particulate component of lakes than in the aqueous fraction [22, 23] (Supplementary Dataset 6; Fig. S5) supports the proposal that cyanobacteria recruit *pepM*-carrying microbes that create a localized phycospheric phosphorus pool through sustainable phosphonate biosynthesis. Further information on the distribution of phosphonate biosynthetic capability in the phytoplankton phycosphere, as well as their expression and regulation pattern, is needed to identify their exact roles.

The role of cyanobacteria in methane biogenesis within oxygenated surface waters, especially in freshwater ecosystems, has long been equivocal, due to the small number of strains capable of methanogenesis [11, 17, 24, 80]. In contrast, heterotrophic bacteria that carry a C-P lyase gene cluster and efficiently release methane through MPn degradation are abundant in cyanobacterial phycospheres [10, 18] and elsewhere in bodies of water [67]. Accordingly, the data presented here strongly suggest that although some cyanobacteria may liberate this greenhouse gas directly [81], the recruitment of phosphonate-metabolizing microbes may be more important in methanogenesis through MPn catabolism.

## Conclusions

Phytoplankton-bacteria interactions are arguably the most significant inter-organism association in aquatic ecosystems [26]. In this work, abundant phosphonate-catabolizing bacteria were found to be active in the phycosphere of cyanobacteria samples from blooms in freshwater lakes and laboratory cyanobacteria cultures. These heterotrophic bacteria components can sustain the proliferation of *Microcystis* in phosphate deficient waters via their ability to metabolize phosphonates that facilitate the maintenance of bloom. The widespread occurrence of phosphonate metabolism and methane release in *Microcystis* bloom water may be explained by dynamic features of cyanobacteria consortia.

## Supplementary Information

**Additional file 1: Fig. S1**. Schematic diagram of *phn* gene clusters in 128 phycospheric heterotrophic bacterial bins. Refer to Supplementary Dataset 3 for detailed strain information. **Fig. S2.** Standard curves of the four qPCR primer sets used in this study. A, 16S rRNA gene; B, PsudmoPJ; C, RhizobPJ; D, RhispiPJ. **Fig. S3.** PafA (A) and AepX (B) protein function validation results in phylogenetic trees. Data used in tree construction are from Murphy et al. [62] and Lidbury et al. [63], respectively. Trees were constructed using maximum likelihood methods under the LG + Gamma + Invariant model. A) Two out of eight potential PafA protein hits (red branches) are located in the main PafA tree (green branches) and their function are confirmed. B) All potential AepX protein hits (red branches) are located out of the main AepX tree (green branches), indicates the mis-annotation of this protein. **Fig. S4.** Sequence similarity network for PhnY* and PhnZ unigenes specificity validation. The method used in network construction was described in Gama et al. [64]. A) The network is based on 1000 unique sequences retrieved from a BLAST analysis of HF130PhnY*. Individual clusters correspond to sequences with >40% identity (alignment score = 60). B) The network is based on 5000 unique sequences retrieved from a BLAST analysis of HF130PhnZ. Individual clusters correspond to sequences with >45% identity (alignment score = 40). Nodes in purple: MPn-specific genes; node in green: 2-AEP-specific genes; nodes in red: unigenes generated with meta-transcriptomic data in this work. **Fig. S5.** Percentage of total organic phosphorous that occurs as phosphonates in different fractions of various sources of freshwater. Data was obtained through a literature search for quantitative measurements of freshwater phosphorous (including phosphonate) contents. Refer to Supplementary Dataset 6 for detail.

**Additional file 2: Dataset 1.** Information for 162 sequenced cyanobacteria strains from FACHB. **Dataset 2.** Genomic information for 647 high-quality phycospheric heterotrophic bacterial bins. **Dataset 3.** Information of 128 high-quality phycospheric heterotrophic bacterial bins that encode C-P lyase gene clusters and their NCBI accession numbers. **Dataset 4.** Information and environmental parameters of bloom water samples used in this study from Taihu Lake and Dianchi Lake. **Dataset 5.** Information regarding 22 phosphonate catabolizing phycospheric heterotrophic bacterial isolates and NCBI accession numbers of 16s rDNA and *phnJ* genes. **Dataset 6.** Reports of phosphonate and phosphate concentrations from previous published studies.

## Data Availability

The sequence of each C-P lyase gene cluster was deposited in NCBI with accession numbers from OK646139 to OK646317. The 16S rDNA and *phnJ* sequences were deposited in NCBI with accession numbers OL798001-OL798022 and OL829851-OL829850. The genome assembles of six phosphonate-degrading phycospheric isolates were deposited in the NCBI Bioproject database under accession number PRJNA949153. The raw metatranscriptome data used in this study were uploaded by Zhu et al. [55] (BioProject: PRJNA664620), and the data were reanalyzed with the authors’ permission. Other data are available on request.

## Abbreviations

MPn: Methylphosphonate
FACHB: Freshwater Algae Culture Collection at the Institute of Hydrobiology
NBRIP: National Botanical Research Institute phosphate growth medium
qPCR: Quantitative polymerase chain reaction
WT: Water temperature
DO: Dissolved oxygen
chl *a*: Chlorophyll *a*
TP: Total phosphorus
TDP: Total dissolved phosphorus
SRP: Soluble reactive phosphorus
TN: Total nitrogen
TDN: Total dissolved nitrogen
