# Supplementary material for: The facilitating role of phycospheric heterotrophic bacteria in cyanobacterial phosphonate availability and Microcystis bloom maintenance
Source: Microbiome. 2023 Jun 26;11:142. doi: 10.1186/s40168-023-01582-2 (PMC10291797; doi:10.1186/s40168-023-01582-2)
Supplement: Supplementary file 2 — Additional file 1: Fig. S1. Schematic diagram of phn gene clusters in 128 phycospheric heterotrophic bacterial bins. Refer to Supplementary Dataset 3 for detailed strain information. Fig. S2. Standard curves of the four qPCR primer sets used in this study. A, 16S rRNA gene; B, PsudmoPJ; C, RhizobPJ; D, RhispiPJ. Fig. S3. PafA (A) and AepX (B) protein function validation results in phylogenetic trees. Data used in tree construction are from Murphy et al. [62] and Lidbury et al. [63], respectively. Trees were constructed using maximum likelihood methods under the LG + Gamma + Invariant model. A) Two out of eight potential PafA protein hits (red branches) are located in the main PafA tree (green branches) and their function are confirmed. B) All potential AepX protein hits (red branches) are located out of the main AepX tree (green branches), indicates the mis-annotation of this protein. Fig. S4. Sequence similarity network for PhnY* and PhnZ unigenes specificity validation. The method used in network construction was described in Gama et al. [64]. A) The network is based on 1000 unique sequences retrieved from a BLAST analysis of HF130PhnY*. Individual clusters correspond to sequences with >40% identity (alignment score = 60). B) The network is based on 5000 unique sequences retrieved from a BLAST analysis of HF130PhnZ. Individual clusters correspond to sequences with >45% identity (alignment score = 40). Nodes in purple: MPn-specific genes; node in green: 2-AEP-specific genes; nodes in red: unigenes generated with meta-transcriptomic data in this work. Fig. S5. Percentage of total organic phosphorous that occurs as phosphonates in different fractions of various sources of freshwater. Data was obtained through a literature search for quantitative measurements of freshwater phosphorous (including phosphonate) contents. Refer to Supplementary Dataset 6 for detail. [file 40168_2023_1582_MOESM1_ESM.pdf]

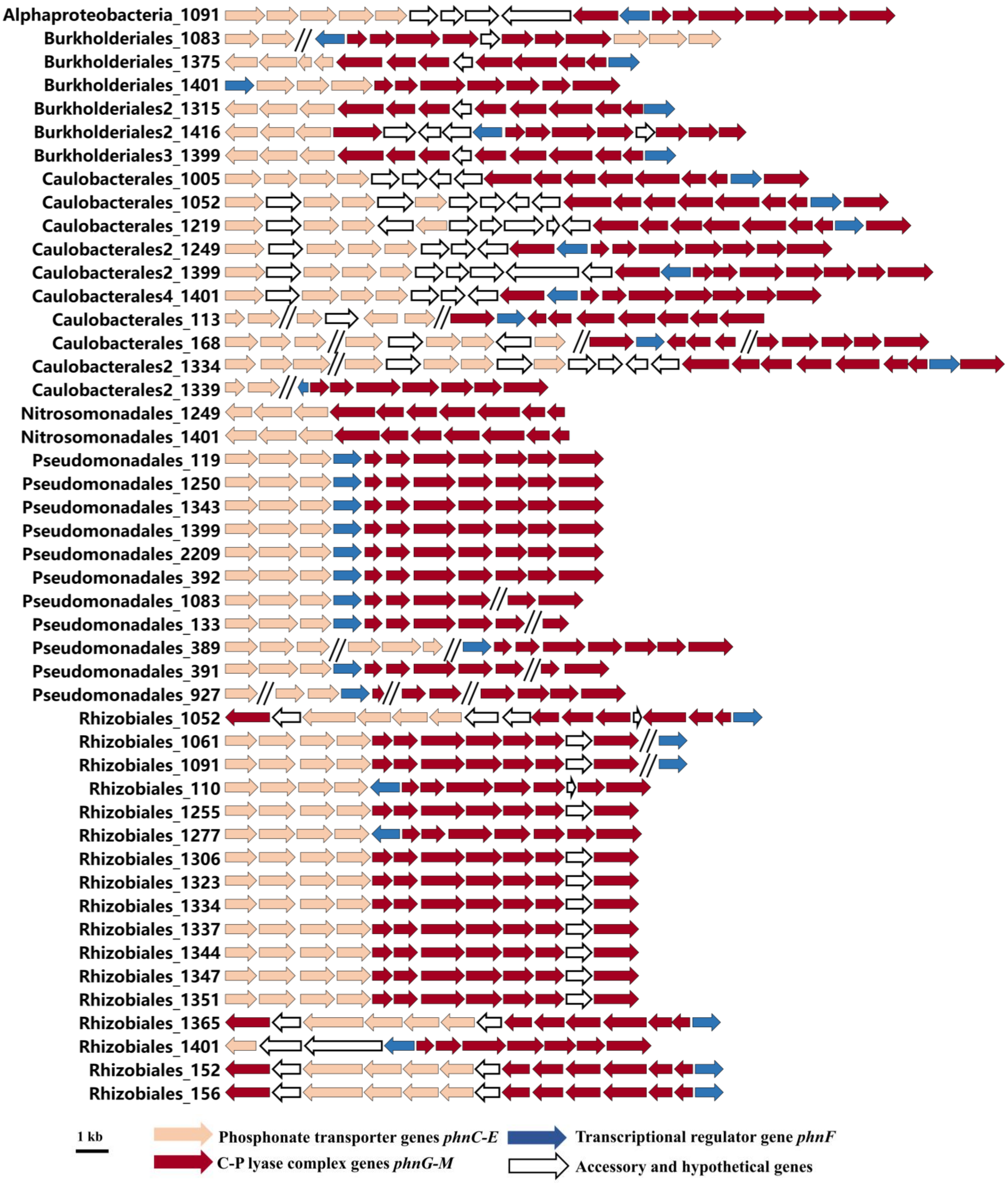


**Fig. S1 Schematic diagram of *phn* gene clusters in 128 phycospheric heterotrophic bacterial binnings .** Refer to Supplementary Dataset 3 for detailed strain information.

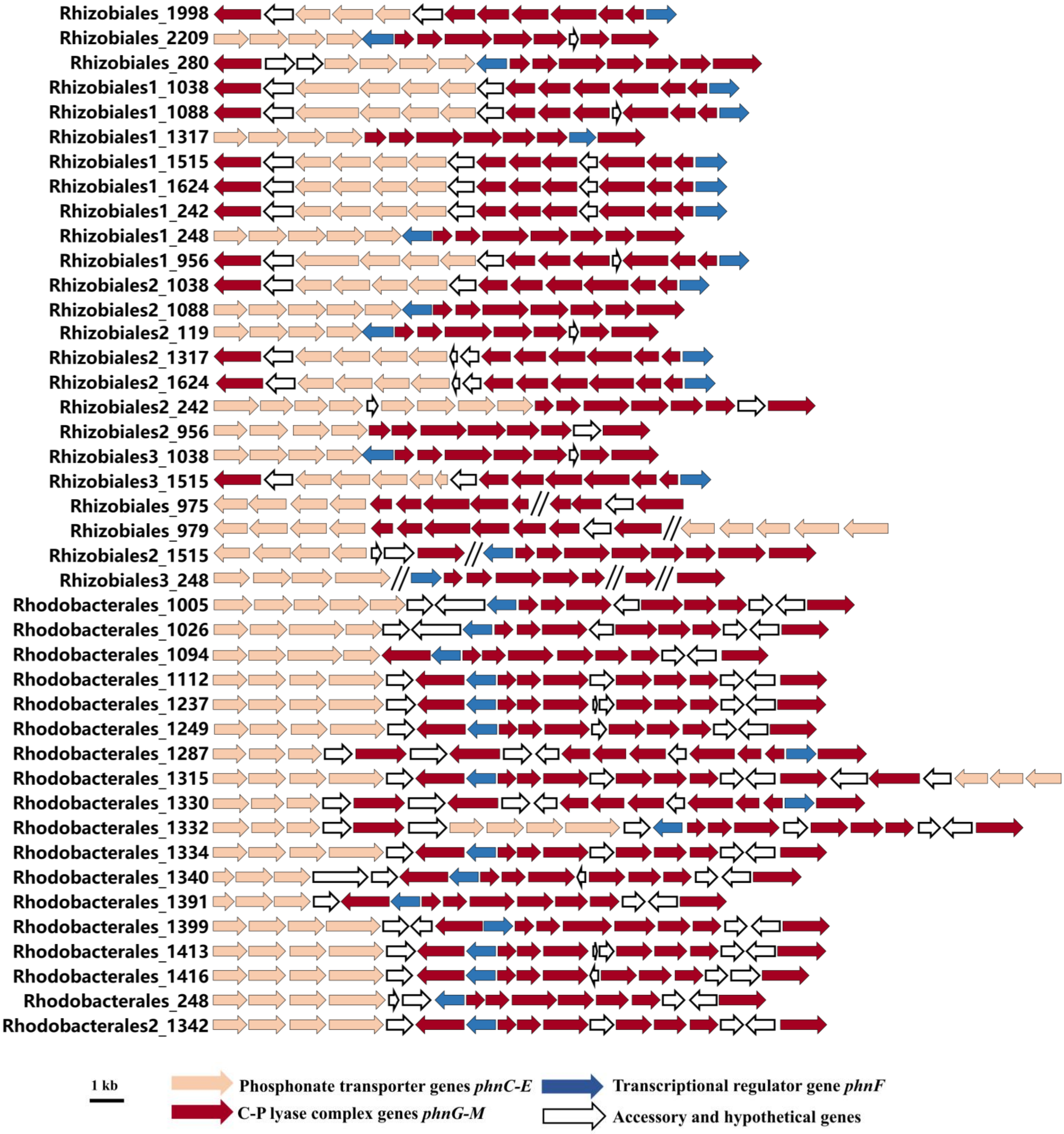


**Fig. S1 Continue**

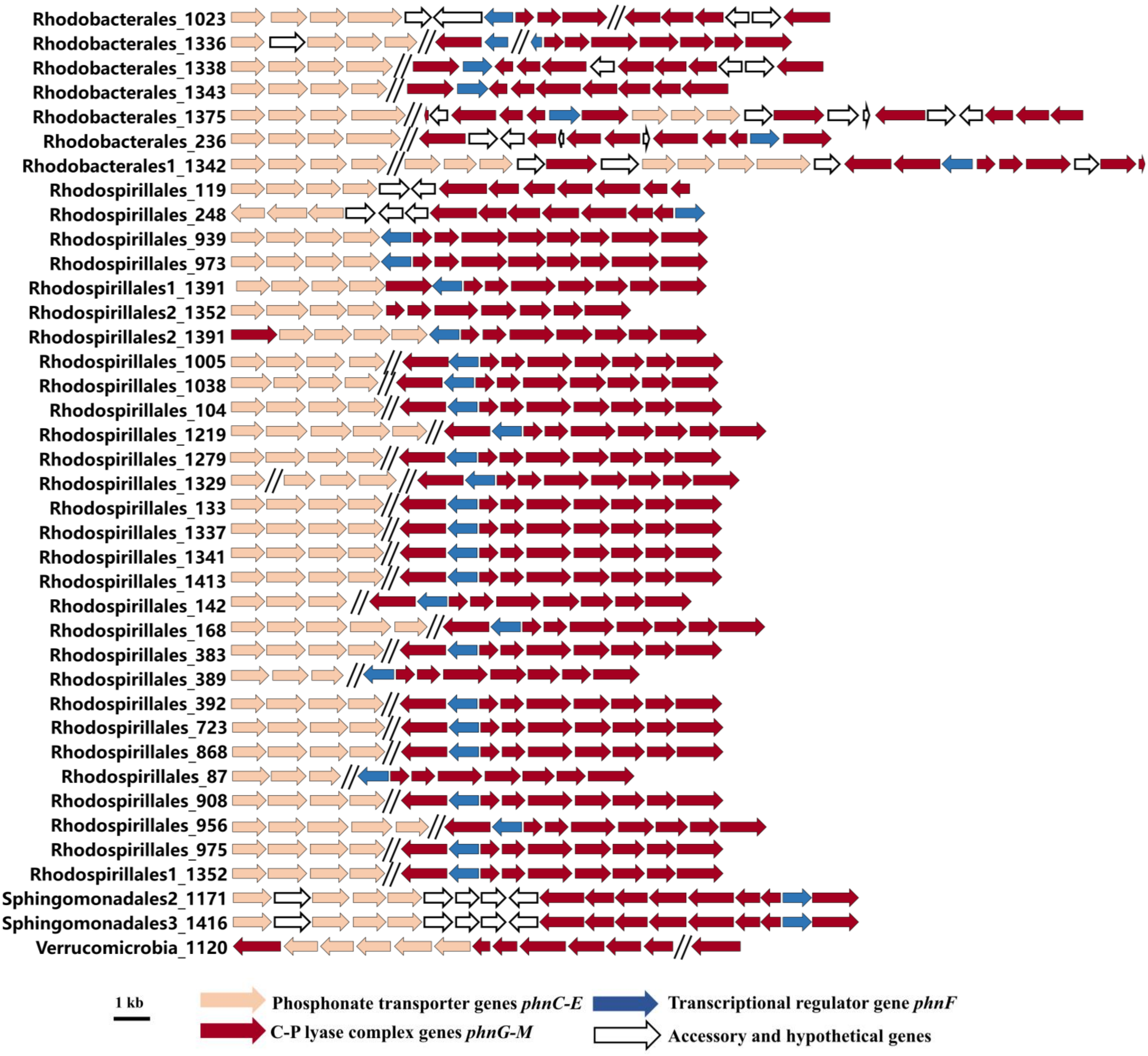


**Fig. S1 Continue**

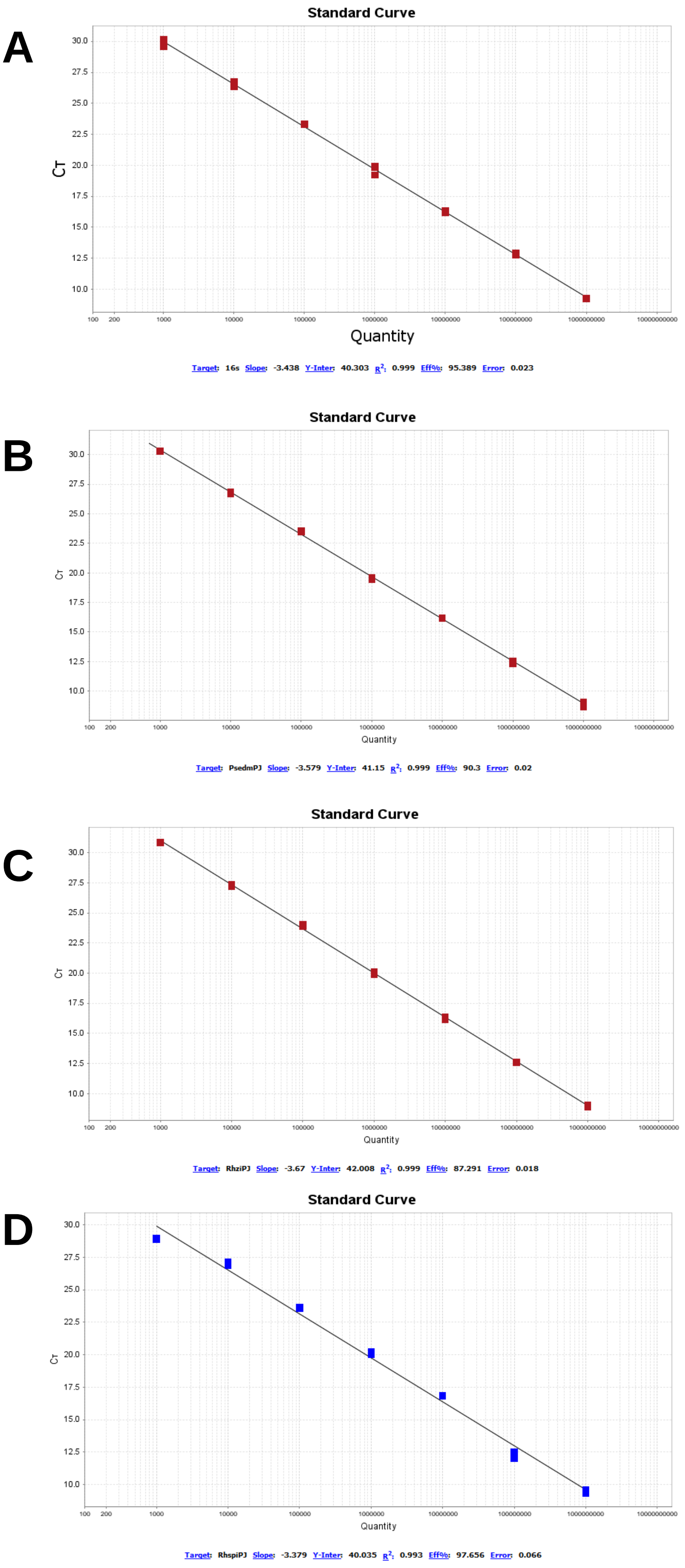


**Fig. S2 Standard curves of the four qPCR primer sets used in this study.**

**A**, 16S rRNA gene; **B**, PsudmoPJ; **C**, RhizobPJ; **D**, RhispiPJ.

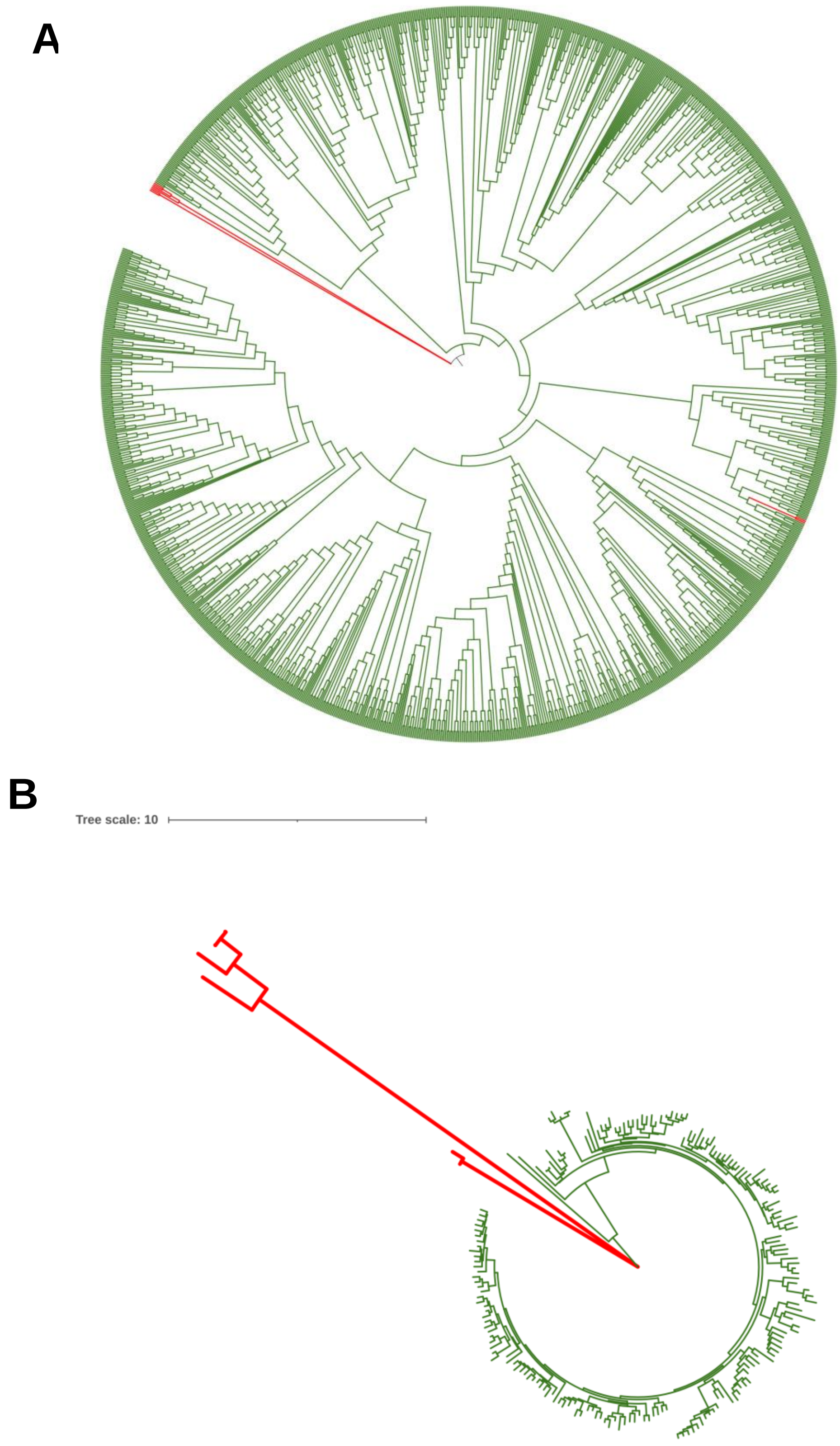


**Fig. S3 PafA (A) and AepX (B) protein function validation results in phylogenetic trees.** Data used in tree construction are from Murphy et al. (2021) and Lidbury et al. (2022), respectively. Trees were constructed using maximum likelihood methods under the LG + Gamma + Invariant model. **A**) Two out of eight potential PafA protein hits (red branches) are located in the main PafA tree (green branches) and their function are confirmed. **B**) All potential AepX protein hits (red branches) are located out of the main AepX tree (green branches), indicates the mis-annotation of this protein.

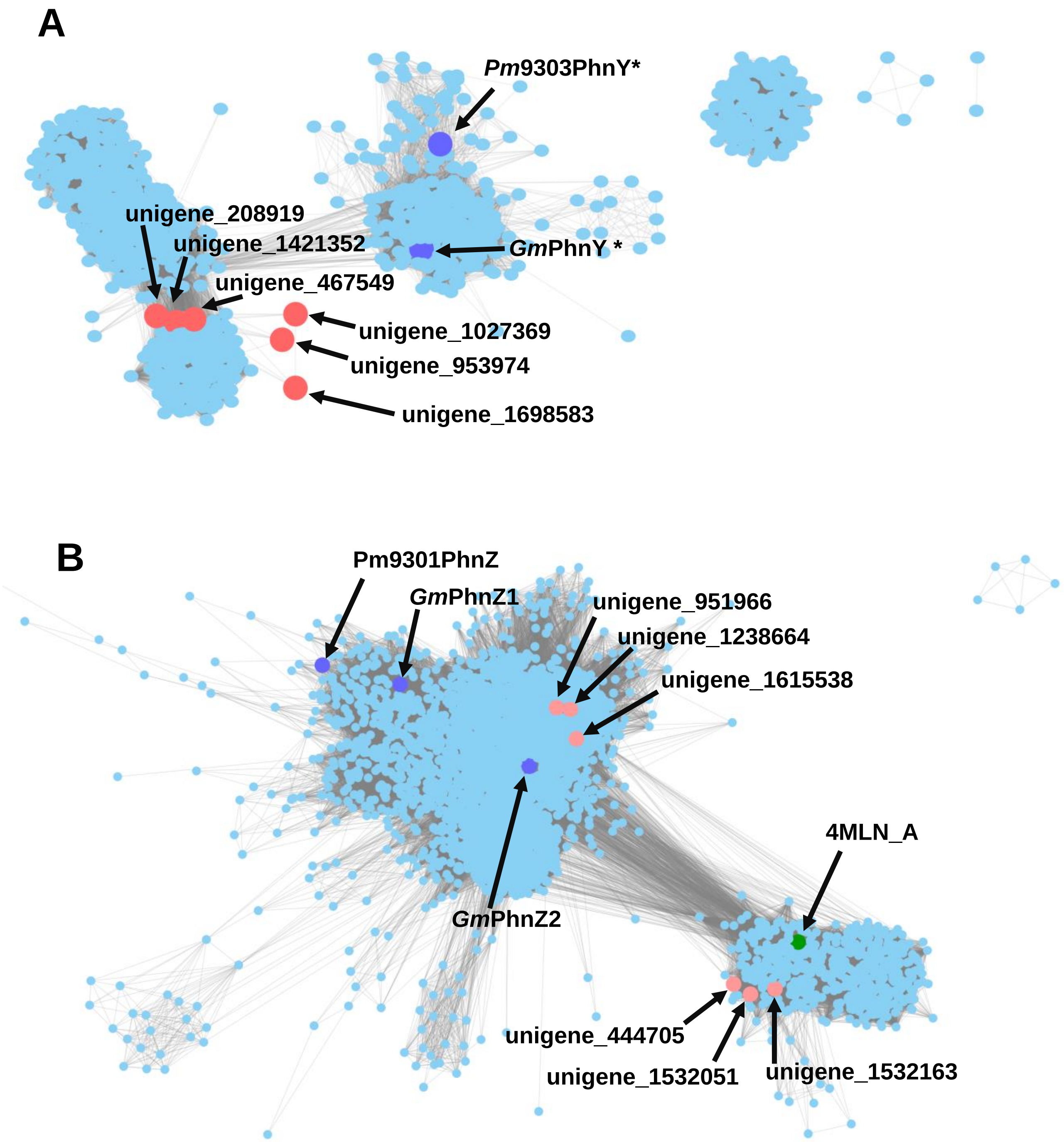


**Fig. S4 Sequence similarity network for PhnY* and PhnZ unigenes specificity validation.** The method used in network construction was described in Gama et al., 2019. **A**) The network is based on 1000 unique sequences retrieved from a BLAST analysis of HF130PhnY*. Individual clusters correspond to sequences with >40% identity (alignment score = 60). **B**) The network is based on 5000 unique sequences retrieved from a BLAST analysis of HF130PhnZ. Individual clusters correspond to sequences with >45% identity (alignment score = 40). Nodes in purple: MPn-specific genes; node in green: 2-AEP-specific genes; nodes in red: unigenes generated with meta-transcriptomic data in this work.

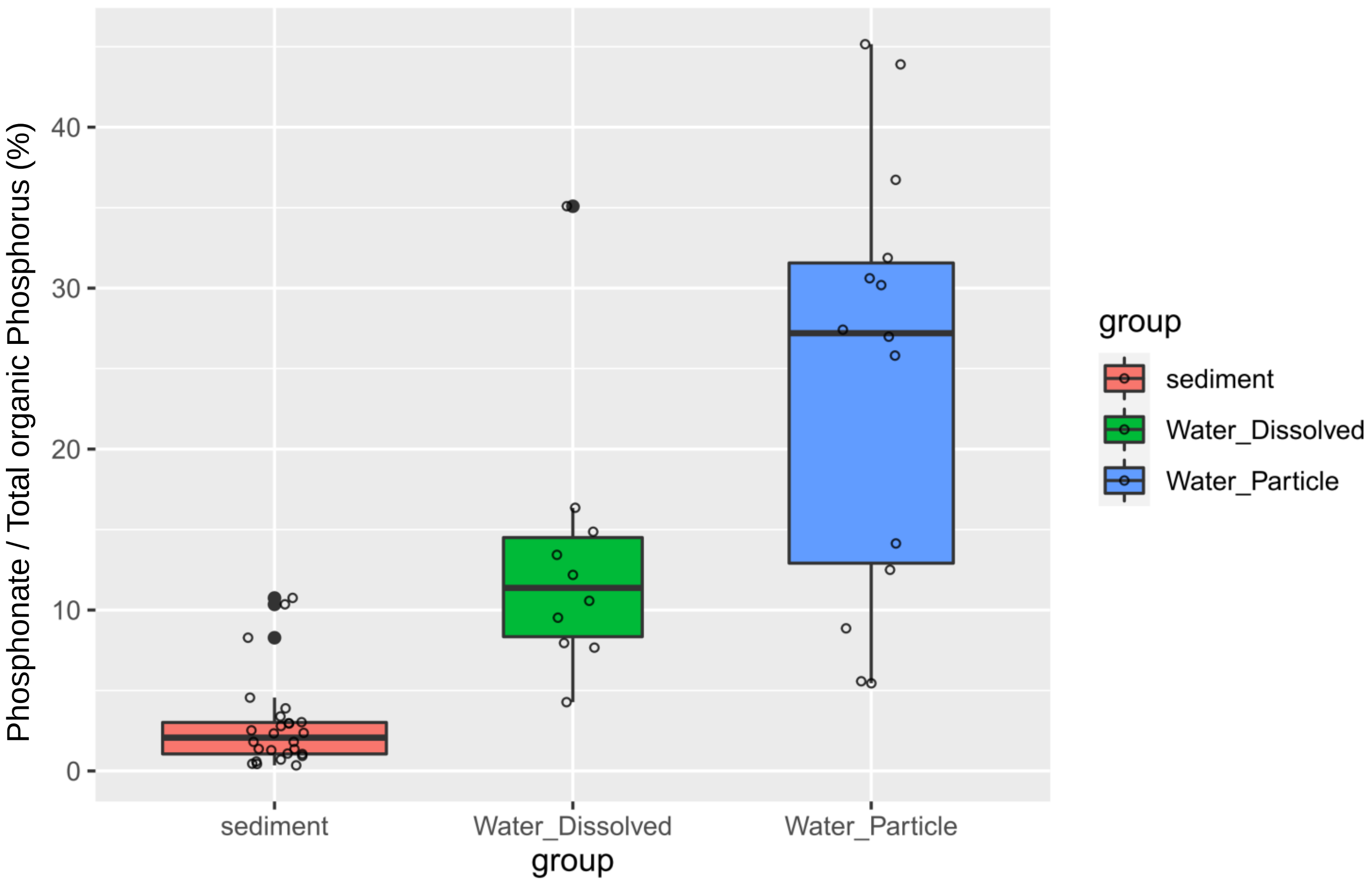


**Fig. S5: Percentage of total organic phosphorous that occurs as phosphonates in different fractions of various sources of freshwater.** Data was obtained through a literature search for quantitative measurements of freshwater phosphorous (including phosphonate) contents. Refer to Supplementary Dataset 6 for detail.
